# “The System Doesn’t Let Us in”—A Call for Inclusive COVID-19 Vaccine Outreach Rooted in Los Angeles Latinos’ Experience of Pandemic Hardships and Inequities

**DOI:** 10.3390/ijerph19105785

**Published:** 2022-05-10

**Authors:** Yelba M. Castellon-Lopez, Savanna L. Carson, Lisa Mansfield, Nanibaa’ A. Garrison, Juan Barron, D’Ann Morris, Ejiro Ntekume, Stefanie D. Vassar, Keith C. Norris, Arleen F. Brown, Alejandra Casillas

**Affiliations:** 1 Department of Family Medicine, UCLA David Geffen School of Medicine, University of California, Los Angeles, CA 90095, USA; 2 Division of General Internal Medicine and Health Services Research, Department of Medicine, David Geffen School of Medicine, University of California, Los Angeles, CA 90095, USA; scarson@mednet.ucla.edu (S.L.C.); lmansfield@mednet.ucla.edu (L.M.); nanibaa@socgen.ucla.edu (N.A.G.); juanbarron@mednet.ucla.edu (J.B.); dmmorris@mednet.ucla.edu (D.M.); entekume@mednet.ucla.edu (E.N.); svassar@ucla.edu (S.D.V.); kcnorris@mednet.ucla.edu (K.C.N.); abrown@mednet.ucla.edu (A.F.B.); acasillas@mednet.ucla.edu (A.C.); 3 Institute for Society & Genetics, College of Letters and Science, University of California, Los Angeles, CA 90095, USA; 4 Institute for Precision Health, Department of Medicine, David Geffen School of Medicine, University of California, Los Angeles, CA 90095, USA; 5 Olive View-UCLA Medical Center, Sylmar, CA 91342, USA

**Keywords:** community engagement, health disparities, COVID-19 pandemic, Latino community, COVID-19 vaccine outreach

## Abstract

Objective. Latino adults in Los Angeles have experienced disproportionate cases, deaths, and socioeconomic impacts from the COVID-19 pandemic. This qualitative study aimed to explore community perspectives on readiness for COVID-19 vaccination and to identify culturally tailored vaccine outreach strategies. Methods. We conducted virtual focus groups with Los Angeles County Latino/a residents via Zoom between December 2020 to January 2021, as the first COVID-19 vaccines were receiving Emergency Use Authorization (EUA). Focus groups were facilitated in Spanish and English by bilingual members of the research team. Discussions were analyzed via Atlas.ti software using reflexive thematic analysis. Results. Three focus groups (*n* = 15; four to six people each; two Spanish focus groups; one English) were conducted. Thematic findings centered on Latino COVID-19 vaccine equity: (1) Disproportionate infection risk due to essential worker status and socioeconomic burdens, misinformation, and familial or cultural tensions (2) Concerns for inequitable vaccine access due to immigration fears and limited healthcare access, and (3) A need for community-centered COVID-19 vaccine outreach and access. Conclusions. Our study on early Latino adult reactions to vaccine roll-out suggests the need for outreach strategies centering on validating community hardships, combating dis-/misinformation through trusted sources, and addressing socio-economic needs impacted by the pandemic.

## 1. Introduction

California is home to the largest population of Latino residents in the US [1], a group that has experienced disproportionate cases, deaths, and socioeconomic impacts from the Coronavirus Disease 2019 (COVID-19) pandemic. As of early 2022, Latino residents continue to make up 48% of infections and 46% of COVID-19-related deaths in California, despite being only 39% of the state’s population [2]. Nationally, US Latinos are twice as likely to be infected with COVID-19 and more than twice as likely to die from COVID-19 than non-Hispanic White peers [3].

Known structural and socioeconomic factors in the home and workplace, which increase COVID-19 risk, are more prevalent among the Latino community. These include employment as essential workers [4], lack of adequate personal protective equipment [5], poor worksite protection or hazard pay, paid sick leave [6], language barriers [7], living in multigenerational family units, crowded housing [8,9,10], and lower rates of remote employment (working from home), among others [11]. These risks are magnified by additional structural barriers that increase Latino residents’ risk of COVID-19 morbidity and mortality, such as poorer access to COVID-19 testing secondary to online scheduling requirements and wait times limiting timely diagnosis and treatment options, and higher prevalence and severity of many common chronic diseases, coupled with decreased health insurance and healthcare service access, leading to worsening infection risk factors [7,12,13,14]. As has also been observed in other historically and contemporaneously under-resourced and disinvested communities, COVID-19 among the U.S. Latino population has demonstrated a need to approach the pandemic recovery through a lens that addresses the social determinants of health [15].

COVID-19 vaccination is the most effective public health strategy to mitigate morbidity and mortality from the virus; however, Latino residents also lag in vaccine uptake across California and the U.S [16]. Recent studies have identified several barriers towards COVID-19 vaccination, and specifically among communities of color. Studies have pointed to medical mistreatment (past/current), misinformation and disinformation, and limited access to vaccination registration and vaccination sites, as some of the persistent barriers in the community [17,18,19,20,21]. U.S. Latino residents, specifically, have pointed to concerns about healthcare costs and lack of health insurance as a vaccine barrier, Spanish-language disinformation regarding the vaccine, and fear of deportation (among the undocumented) when seeking vaccination [22,23,24,25,26]. Latino residents have also expressed emotional distress and generalized worry related to the pandemic’s substantial economic impact (which may be a detractor from prioritizing their vaccination), given the community’s disproportionate representation in the labor markets that have been hardest hit by the pandemic’s business slow-downs and closures (i.e., restaurants, domestic work, caregiving) [23,27].

The purpose of this qualitative study was to explore early reactions and readiness for COVID-19 vaccination, and understand perceived facilitators and barriers to vaccination among Latino residents of Los Angeles County, as COVID-19 vaccines were being approved for emergency use authorization (EUA) in December 2020 [28]. The goal was to learn from these early community views to develop tailored strategies that would advance COVID-19 vaccine equity in Latino communities.

## 2. Materials and Methods

### 2.1. Design and Study Setting

This study was nested within a larger qualitative study on COVID-19 vaccine hesitancy among multiethnic communities in Los Angeles County [18]. We used the Vaccine Hesitancy Matrix (VHM), developed by the World Health Organization Strategic Advisory Group of Experts on Immunization Vaccine Hesitancy Working Group, to categorize prominent themes across various racial and ethnic groups in the larger parent study reported elsewhere [18]. The VHM provides a framework for the multifaceted nature of vaccine acceptability including individual and personal perception of the vaccine, group or social influences, and contextual influences such as historical, sociocultural, health system/institutional, and economic factors, and their interconnected role in shaping vaccine decision making [29,30,31]. For this Latino-specific project, we conducted three virtual focus groups with Los Angeles County Latino/a adults from 16 December 2020, to 28 January 2021, via Zoom [21]. Individuals were recruited through Los Angeles Latino organizations, with focus groups on COVID-19 information being promoted at virtual town halls, hosted by these community organizations. We intentionally recruited for essential workers and those who resided in a low-income zip code (median household income < $40,000, 2010 U.S. Census), given the likely higher risk for COVID-19 infection [19,22,23]. Each focus group lasted about 60–90 min and was conducted in English or Spanish. We organized focus groups by age (<50 years and >50 years) to include generational perspectives. The UCLA Institutional Review Board approved the study. We report our findings using the Standards for Reporting Qualitative Research [24].

### 2.2. Data Collection and Focus Groups Guide 

The focus group discussion guide was based on prior studies of general vaccine hesitancy in racial and ethnic minority communities, and modified based on discussion with our Los Angeles community partners [28]. The guide included questions on concerns, risks, benefits, information sources, trusted entities, barriers, and recommendations for improving COVID-19 vaccine access (Table 1). Participants were asked to express their thoughts and opinions as individuals and as “community experts,” and were encouraged to share what they had heard discussed among their families and communities. Bilingual Latina physicians facilitated the focus groups (YCL, AC). All sessions were recorded, transcribed, and translated into English. Spanish translations were checked for accuracy by the bilingual facilitators. Participants completed an online demographic survey and received a $100 gift card for their time.

### 2.3. Analysis

As described in the larger study [18], transcripts were analyzed using Atlas.ti qualitative software using reflexive thematic analysis [32,33,34]. Two experienced coders (SLC, LNM) reviewed the transcripts and field notes to develop a preliminary codebook, then tested and amended the codebook’s initial practicality following the coding of two transcripts. The coders reached an iterative consensus on the evolving codebook, code definitions, and coding approach, and used memos to document thematic evolution throughout the analysis. Triangulation was achieved by reviewing the field notes and holding iterative discussions with all moderators/facilitators. Preliminary results were validated with key community stakeholders in community partnered meetings. Results herein focus on salient Latino/a-specific considerations for vaccine confidence and accessibility, not previously described in the larger multiethnic study manuscript [18].

## 3. Results

### 3.1. Participant Demographics

Three Spanish or English focus groups of four to six people each (*N* = 15) were conducted (*N* = 48 peopled screened, *N* = 19 scheduled to participate). Most participants resided in low-income zip codes (73%). About half described themselves as essential workers (53%). Less than half (40%) reported they were “likely or very likely to receive the vaccine when it became available.” Of those who would be willing to receive a vaccine, the primary reasons for vaccination were to keep family (93%), their community (80%), and themselves (80%) safe. Demographics and survey responses are shown in Table 2 and Table 3. 

### 3.2. Themes

In terms of the discussions with participants, overarching themes include: (1) Identification of disproportionate infection risks due to essential worker status and socioeconomic burdens, misinformation, and familial or cultural tensions, (2) Concerns for inequitable vaccine access due to immigration fears and limited healthcare access, (3) A need for community-centered COVID-19 vaccine outreach and access. Below we discuss each theme in the context of illustrative participant quotations and subthemes that highlight important nuances.

#### 3.2.1. Identification of Disproportionate Infection Risk Due to Essential Worker Status and Socioeconomic Burdens, Misinformation, and Familial or Cultural Tensions

(1) Heightened COVID-19 Vulnerability Due to the Essential Worker Status, Economic Instability

Participants described the disparate amount of burdens Latino essential workers have faced due to hardship in the pandemic. One participant explained:

*“The truth is that people haven’t stayed at home. They have to go to work. They have to go out to get their food. I believe that the area that has been hit the hardest has been the Latino community for that reason because they have to go to work … that’s the reason for the high number of people that have been infected.”*

Others echoed this type of distress: “*I feel that all the Latino families are suffering. It’s very painful. I feel it in my heart. It’s terrible in every way, spiritually, economically. Latino families are not equipped; they are going through hard times.”* Another participant described the Latino culture as working hard to support basic needs for all and how this impacts the risk of infection, stating, *“I recognize the Latino community has a great spirit of unity, and of hard work and responsibility, and, many times, our responsibility leads us to make some mistakes.”*

Participants not only described their personal experience with the COVID-19 pandemic impact, they also expressed empathy and advocated for the needs of Latinos in more extreme poverty and those who are marginalized, explaining, those “*whose economic level is low… they are more vulnerable and can be seriously affected. In fact, statistics show that the lower your economic level is, the harder the virus attacks*.”

(2) Propagation of Dis- and Misinformation in Latino Communities and the Media

Misinformation was described as a prominent obstacle to vaccine knowledge and acceptability. Participants described how various news sources, including social networks, family, Spanish-language media, and news sources from Spanish-speaking and/or Latin-American countries, affected community-propagated misinformation. One participant explained how:

*“The Spanish speaking channels like Telemundo, Univision is where the Latino population get the information; or, from the social networks that include personalities from their own countries, who…tell people things that are not true and people have the tendency to culturally follow the trends of what is in happening in their countries. So, that would be an entry point for false information here in Los Angeles … So, sadly, [the Latino media] doesn’t inform, they rather disinform.”*

Participants described how the conflicting information circulated, explaining “*in the Latin community there are so many myths that are going around*” and how sorting through the various news was difficult as another participant stated, “*The reality is that all of us have too much information… right now the information is chaotic*.”

(3) Familial or Cultural Ties in Conflict with COVID-19 Prevention Strategies

Participants described how multi-generational households had difficulty adapting to the recommended COVID-19 social distancing guidelines due to household density or cultural behaviors, potentially increasing the COVID-19 transmission risk. A participant explained how Latino familial structures could sometimes fall in to conflict with COVID-19 risk mitigation.

*“As Latinos, we don’t like to make people feel bad by telling them that you don’t want them inside your house… when someone comes to visit, it’s your sister or your brother… the truth is that then you feel you’re going to get sick, the whole family will get sick.”*

Other participants described sociocultural difficulties in navigating familial risk including how separating from family members and elders could be seen as abandonment.

#### 3.2.2. Concerns for Inequitable Vaccine Access Due to Immigration Fears and Limited Healthcare Access

(1) Collective Awareness of Impending Vaccine Inequity Related to Migration Status

Participants expressed concerns about unequal access to vaccination through government entities, systematic difficulties in vaccine registration, and documentation requirements for vaccine registration, particularly as it related to undocumented immigrant populations. One participant questioned, “*The government is not talking about vaccination for everybody. It’s talking about vaccination phases. First phase, second phase. So, which phase are undocumented people going to be in*?”

Another participant worried about undocumented populations obtaining vaccination and explained, *“I don’t know what the government is thinking, at times I think they are prioritizing vaccinating the population in the United States with citizenship or green cardholders. Since that population hasn’t been vaccinated yet … what are they going to do with the millions of undocumented people, I don’t know how there are? They should also be covered … There shouldn’t be excuses like, ‘I’m not going to give you the vaccine because you lack the proper paperwork.’ We’ll have to fight. And there will be social fighting where we’ll be the main actors to promote a vaccination plan for everybody. That will be the subject’s name, vaccination for everybody. That will be the most critical point, I think.”*

(2) Concerns for Vaccine Inequity Due to Poor Access to Healthcare

Participants described longstanding inequities in health access among Latinos, such as the lack of health insurance in their communities, and fear of public charge. They worried how these would lead to unequal access to the vaccine. One person explained how past experiences with poor healthcare access and high cost affect their perceptions of COVID-19 care: “*People think they need to have insurance to get tested for COVID… People think they are going to be charged, so they must have insurance… A neighbor just died because he had no insurance, and he stayed there because the doctor was going to be very expensive.”*

Fears of being deemed a public burden due to use of public benefits resulting in denial of a green card, visa, or legal admission into the United States, or “public charge,” brought additional concerns for healthcare access, “*undocumented people are afraid to go to the doctor as if they were forbidden to do that for fear that they are a public burden.”* Another participant explained, “*in California we have… I don’t know how many millions of people there are without access to a health care insurance. The reason? They don’t have the proper documentation, and that will be a huge barrier because many people don’t have medical coverage, and therefore, they don’t have health control. Those people will be more vulnerable to get the vaccine*.”

#### 3.2.3. A Need for Community-Centered COVID-19 Vaccine Outreach and Access

Participants described community engagement and emphasized that equity could be more feasibly achieved by language translation, culturally-specific media, personal interaction with Latino health professionals to dispel myths, and in-person outreach like door-to-door campaigns, and with vaccines and information brought directly into Latino-heavy neighborhoods. One participant further explained the need for medical professional interaction in communities, “*you really don’t have, like, liaisons or anything like that from the medical field that are going into communities and talking to people on a personal level about anything, really, much less COVID or the vaccine*”.

Participants described how to leverage trusted entities to engage and create “unity” in the community for vaccination. One participant recommended outreach through “*community groups around the county that reach certain groups of people in the communities directly and that people trust them, for example, [local community-based organizations] … the churches, the schools, [so] those of us who are going to take the vaccine, feel confident*.” Another participant explained the need to build trusted relationships within communities over time through outreach describing how “*especially if it’s somebody that you saw quite regularly… somebody that you became familiar with that you know that you can trust*”.

Another participant stated the importance of engagement that could cater towards questions that were common among community members, “*I think it would help if they had maybe like workshops for the community… with doctors and just different people from different fields kind of explaining every bit of everything that’s going on … where people can ask any question they have, anything from what is COVID to how does the vaccine work or anything like that. Just kind of a very freeing workshop where you’re not being kind of forced to do anything … It’s just any questions you have bring them to the table, we’ll answer them”.*

Participants described frustration with systemic delays in vaccination access for their communities. Many participants expressed a need for vaccination navigation due to registration difficulties and fears of inequity (as described above). A participant relayed how when trying online registration, “*the system doesn’t let them in,*” and another participant told how some “*haven’t been lucky enough to be able to do it [vaccination registration]. I mean, neither by the link nor by the phone… Yesterday, I spent three, four hours waiting, and nothing happened*.” Participants described strategies for increasing vaccine access, suggesting how community-based strategies can reduce fears and increase access. A participant suggested.

*“Mobile clinics that would come out to the most vulnerable, homeless, undocumented, no insurance, for their regular checkups… for those that don’t have transportation to go to the mega-sites to get vaccines… And before getting vaccinated, maybe having some type of, like, orientation, like, these are side effects, answer a few questions that people might have and then take it from there. I think they feel safer instead of going into a big building, you know, these are mobile units. They feel like, okay, it’s less questions, less paperwork, it’s the basic information. They don’t sit and ask for your documentation, so I think it makes people feel safer”.*

## 4. Discussion

Our findings provide insight into the awareness of the disproportionate impact of COVID-19 on Latino communities and key insights as to how vaccine equity could be better achieved in Los Angeles just as COVID-19 vaccines were receiving approval in December 2020 and early 2021. Participants recognized undue risks in their homes and communities due to employment conditions/roles, limited healthcare access and COVID-19 care, immigrant/documentation status, and poor access to culturally and linguistically relevant trusted sources of information to address prominent concerns about the COVID-19 vaccine side effects, and other circulating myths. Latino participants demonstrated a community consciousness and desire to protect and advocate for the most vulnerable, including undocumented immigrants, essential workers, and low-income families. These discussions suggest that outreach for COVID-19 vaccination will need to recognize and address these potential inequities as a salient component of authentic and trustworthy vaccine messaging and access among Latinos.

This study suggests that COVID-19 vaccination protocols should be reassessed to determine whether they contribute to structural access barriers. For example, even asking for identification during vaccination appointments is problematic and creates fear and marginalization [35,36]. These incidents create inequities, and thus public health systems must ensure that these systemic barriers are immediately addressed, and also that people feel confident in getting vaccinated (i.e., trusted messengers that address culturally specific concerns like these), regardless of immigration documentation or insurance coverage.

Participants recommended strategies for COVID-19 vaccine outreach centered in their local community, use of translation services or Spanish-language materials, and messaging from trusted community members and entities. These findings coincide with other studies exploring COVID-19 vaccine acceptance and access among Latinos [24,37]. There were specific calls for the improved delivery of vaccine information including dispelling vaccine myths, discussing possible vaccine side effects through increased access to Latino/a doctors as trusted messengers, and for reducing structural barriers faced at the start of vaccine roll-out like only scheduling vaccine appointments online, and decentralization of vaccine sites and vaccine information from highly resourced communities to their neighborhoods. Participants discussed the pressure to maintain familial and cultural norms that conflicted with COVID-19 social distancing precautions, throughout the pandemic. These findings suggest that to engage the Latino community, COVID-19 information and vaccine outreach should include leveraging trusted community organizations and tailoring public health messaging that emphasizes dedication, commitment, and loyalty to family or “familismo” as reasons for vaccination, which has already been documented in the recent literature as one culturally-pointed strategy to improve COVID-19 messaging and vaccine information among Latino communities [38,39].

Future efforts should also focus on regulating misinformation or disinformation from Spanish language media sources, including those from international sources and Latin America—which one participant alluded to as “chaotic” information [40,41,42,43]. Some Spanish-speaking Latinos may continue to rely on the information on social media from their native country or Latin America to shape their understanding of COVID-19 vaccines because it is delivered in their primary language. Community members shared that social media videos are rapidly disseminated, making it difficult to discern trusted sources from disinformation. This rapid-fire “culturally targeted” content can be misleading and contributes to confusion and mistrust. Our findings also suggest that, culturally congruent medical doctors, community health workers (promotoras), and other community leaders considered trusted sources of information can counteract disinformation by providing evidence-based recommendations and dispelling circulating myths and misconceptions that have gone “viral”.

Using the results from this study as part of the more extensive study in multiethnic groups, additional themes and policy suggestions brought up by Latino and other multiethnic participants were described more in detail [18]. As part of a national outreach effort funded by the NIH Community Engagement Alliance (CEAL) against COVID-19 to address community needs for information about COVID-19 from trusted messengers, we developed a series of English and Spanish-language Latino-focused COVID-19 webinars and town hall meetings on Zoom or WhatsApp by members of the study team. We anchored vaccine messaging around the disproportionate impact of COVID-19 on Latinos. Specifically, we created vaccine messaging that was less prescriptive like “you should get the vaccine” and instead rooted our vaccine recommendation in promoting health equity “it is your right to be vaccinated after all you have endured”. These sessions created space for community dialogue about the traumas experienced during the pandemic, and leveraged lessons learned about vaccine myths and misinformation, addressing these directly in the sessions, destigmatizing people’s confusion and normalizing the need to speak up about misleading information. As of January 2022, we have delivered 40 webinars to 24 community organizations and schools, reaching over 1000 participants (the majority in Spanish) [44,45]. These findings also informed California’s Get Out the Vaccine (GOTV) Campaign [46], a UCLA partnership with the Office of the California Governor and community-based organizations (CBOs) across the State: working as community canvassers to walk door-to-door in some of the COVID-19 hardest-hit neighborhoods across California (following COVID-19 safety protocols) and registering eligible residents for vaccination while connecting people to local resources for food, rent relief, and legal assistance.

This study has limitations, including our small sample size and the time period when the focus groups were collected, which limits the generalizability of the results. We recognize that Latinos are a heterogeneous population, and therefore the perspectives and experiences reported here may not encompass the experiences of all communities. Our study is limited in exploring the intersectionality of demographic characteristics (e.g., level of education, age, gender, occupation) on COVID-19 vaccine acceptance. Future research is needed to explore the intersectionality of demographic characteristics on COVID-19 vaccine acceptability and decision making in the Latino community. Also, focus groups were conducted during the initial phase of the vaccine roll-out in Los Angeles, a county with a strong safety net during a period of rapid adaptations. Our goal was to capture participant feedback and then work with local policymakers, health agencies, and health plans to inform subsequent outreach efforts. Despite these limitations, the study provides critical insights on the hardships caused by the pandemic and suggestions for a path towards recovery from the perspective of the Latino community members.

## 5. Conclusions

The COVID-19 pandemic further deepened the health and social inequities that have plagued our historically and contemporaneously under-resourced and disinvested communities in the US. Counteracting the profound impact and sequelae of the pandemic on the Latino community will require public health initiatives that are tailored to the experiences and perceived needs of this community, and outreach that is visibly familiar with the “Latino pandemic story”, and gives a voice to their lived narrative during this crisis. Moving forward, policies must also support community-centered outreach that addresses social determinants of health and ongoing and underlying health disparities, as the primary framework for achieving COVID-19 vaccine equity for the Latino community.

## Figures and Tables

**Table 1:** Focus Group Question Guide.

Sample Focus Group Questions
‣ Icebreaker: Please state your name, tribal affiliation (if applicable), current feelings on the pandemic, and one word to describe your racial/ethnic community. ‣ What have you or members from your community heard about any vaccines to protect against COVID-19? ‣ What concerns do you, your family, or your community have about receiving the COVID-19 vaccine? What additional information do you need to feel comfortable to receive the COVID-19 vaccine? ‣ When a COVID-19 vaccine is available, who from and where would you feel most comfortable getting the vaccine? ‣ What do you think are some risks and benefits of the COVID-19 vaccine? ‣ Situational questions: It could be the case that some of the vaccines offered may not 100% protect against COVID-19 infection. The vaccine may lower the chances of being infected by COVID-19. Or, if you do get COVID-19, the vaccine may lower your chances of getting very sick from it (reduce the severity of the disease or reduce additional health complications). However, it may not be perfect, and it may not prevent 100% of people from COVID-19. How would you feel about the information (that getting the vaccine does not 100% protect against being infected)? ‣ What challenges may you, your family, or people you know face in getting the COVID-19 vaccine? ‣ What are some ways to get the COVID-19 vaccine to the people who need it most when it becomes available?

**Table 2:** Latino Focus Group Participant Demographics.

Participant Demographics (*N* = 15)	
	No. (%)
Mean age (SD)	41 (10.4)
20–34	2 (13.33)
35–49	6 (40.00)
50–64	5 (33.33)
65+	2 (13.33)
Gender	
Female	10 (66.67)
Male	5 (33.33)
Other	0 (0.00)
Number of minors in the household	
Some High School	5 (33.33)
High School graduate/GED	2 (13.33)
Associate’s/technical degree	5 (33.33)
Bachelor’s degree	2 (13.33)
Graduate degree	1 (6.67)
Prefer not to answer	0 (0.00)
Number of people in household, mean (SD)	3.93 (1.59)
Employment status	
Full time	2 (13.33)
Part-time	5 (33.33)
Unemployed	7 (46.67)
Retired	1 (6.67)
Essential worker	8 (53.33)
Resides within a low-income zip code (Median household income < $40 K, per U.S. Census 2010)	11 (73.33)
Very important or important for all people in community to receive the COVID-19 vaccine	15 (100.00)
Very likely or moderately likely to get an approved COVID-19 vaccine when available	6 (40.00)
COVID-19 history	
I have previously tested positive for COVID-19, I believe I had COVID-19, or had COVID-19-like symptoms	3 (33.33)
No, no symptoms of COVID-19	12 (80.0)
Unsure	0 (0.00)

GED—General Equivalency Diploma; COVID-19—Coronavirus Disease 2019.

**Table 3:** Latino Focus Group Participant Survey.

Reasons for and against Obtaining Vaccination (*N* = 15)	
	No. (%)
Top reasons obtaining a COVID-19 vaccine (check all that apply)	
I want to keep my family safe	14 (93.33)
I want to keep my community safe	12 (80.00)
I want to keep myself safe	12 (80.00)
I want to feel safe around other people	10 (66.67)
I believe life won’t go back to normal until most people get a COVID-19 vaccine	11 (73.33)
I don’t want to get really sick from COVID-19	9 (60.00)
I have a chronic health problem, like asthma or diabetes	2 (13.33)
My doctor told me to get a COVID-19 vaccine	3 (20.00)
Other	0 (0.00)
N/A	0 (0.00)
Top reasons for not obtaining a COVID-19 vaccine (check all that apply)	
I’m concerned about side effects from the vaccine	10 (66.67)
I don’t know enough about how well a COVID-19 vaccine works	6 (40.00)
I don’t trust that the vaccine will be safe	7 (46.67)
Other	4 (26.67)
I don’t want to pay for it	4 (26.67)
I don’t think vaccines work very well	1 (6.67)
I’m not concerned about getting really sick from COVID-19	1 (6.67)
I don’t believe the COVID-19 pandemic is as bad as some people say it is	2 (13.33)
I’m allergic to vaccines	0 (0.00)
I don’t like needles	2 (13.33)
N/A	0 (0.00)

COVID-19—Coronavirus Disease 2019.

## Data Availability

Not applicable.
